# Novel Antimicrobial Indolepyrazines A and B from the Marine-Associated *Acinetobacter* sp. ZZ1275

**DOI:** 10.3390/md17020089

**Published:** 2019-02-01

**Authors:** Komal Anjum, Sidra Kaleem, Wenwen Yi, Guowan Zheng, Xiaoyuan Lian, Zhizhen Zhang

**Affiliations:** 1 College of Pharmaceutical Sciences, Zhejiang University, Hangzhou 310058, China; komalazam@ymail.com (K.A.); 0818331@163.com (G.Z.); 2 Ocean College, Zhoushan Campus, Zhejiang University, Zhoushan 316021, China; kaleemsidra85@yahoo.com (S.K.); 18790658213@163.com (W.Y.)

**Keywords:** *Acinetobacter* sp. ZZ1275, Indolepyrazines A and B, Antimicrobial activities

## Abstract

Two new alkaloids indolepyrazines A (**1**) and B (**2**) were isolated from the marine-derived *Acinetobacter* sp. ZZ1275. Their structures were elucidated through extensive nuclear magnetic resonance (NMR) spectroscopic analyses, high resolution electrospray ionization mass spectroscopy (HRESIMS) data, and electronic circular dichroism (ECD) calculation. Indolepyrazine A represents the first example of alkaloids with an indole-pyrazine-oxindole skeleton. Both **1** and **2** showed antimicrobial activities against methicillin-resistant *Staphylococcus aureus*, *Escherichia coli*, and *Candida albicans* with minimum inhibitory concentration (MIC) values of 12 μg/mL, 8–10 μg/mL, and 12–14 μg/mL, respectively.

## 1. Introduction

Species of the genus *Acinetobacter* are Gram-negative bacteria and important soil organisms. They are also found in hospital environments as a major source of infection in debilitated patients. The species *Acinetobacter baumannii*, in particular, is known to cause nosocomial infections, such as endocarditis, meningitis, pneumonia, septicaemia, and tract infection [1]. Some *Acinetobacter* bacteria were reported to produce secondary metabolites, mainly including siderophores acinetobactin [2], acinetoferrin [3], baumannoferrins A and B [4], polysaccharides [5,6,7], antibacterial succinamide conjugate diacid [8], antifungal volatile compounds [1], and antimicrobial diketopiperazines cyclo-(proline-leucine) and cyclo-(proline-tyrosine) [9]. However, it is our understanding that no natural product has yet been reported from marine-derived *Acinetobacter* species.

In this study, a marine bacterium *Acinetobacter* sp. ZZ1275 was isolated from a sample of coastal muds during the process of our ongoing research to discover novel bioactive compounds from marine-associated microorganisms [10,11,12,13,14]. A crude extract from a scale up culture of the strain ZZ1275 showed activities against the growth of methicillin-resistant *Staphylococcus aureus* (MRSA), *Escherichia coli*, and *Candida albicans*. Chemical investigation of this antimicrobial extract resulted in the isolation and identification of two new alkaloids, named indolepyrazines A (**1**) and B (**2**). Indolepyrazine A is the first report of an indole-pyrazine-oxindole alkaloid and both **1** and **2** had antimicrobial activities. Herein, we have reported the isolation and culture of the strain ZZ1275 as well as the isolation, structural elucidation, and antimicrobial evaluation of the two compounds.

## 2. Results and Discussion

**Figure 1:**
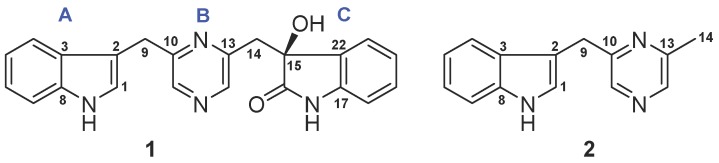
Structures of indolepyrazines A (**1**) and B (**2**).

Based on the result of the 16S rDNA sequence analysis (Supplementary materials, Table S1 and Figure S1), the strain ZZ1275 (Figure S2) was assigned as *Acinetobacter* sp. ZZ1275. The crude antimicrobial extract prepared from a 65 L culture of the strain ZZ1275 was fractionated by column chromatography, followed by purification with high performance liquid chromatography (HPLC), to give compounds **1** and **2** (Figure 1).

**Figure 2:**
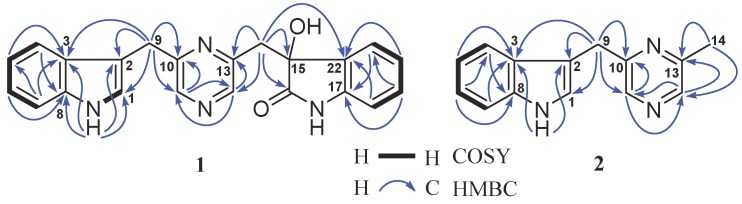
^1^H-^1^H COSY (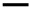) and Key HMBC (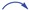) correlations of indolepyrazines A (**1**) and B (**2**).

**Figure 3:**
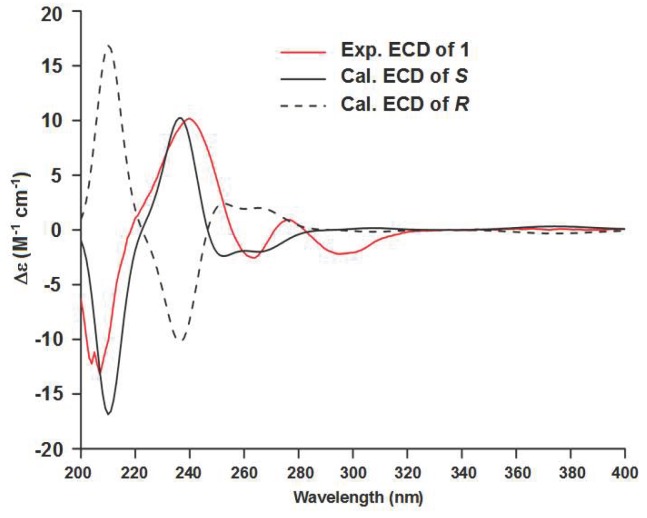
Experimental and calculated electronic circular dichroism (ECD) spectra (200–400 nm) of the model molecules of **1** and indolepyrazine A (**1**) in MeOH.

Compound **1** has a molecular formula of C_22_H_18_N_4_O_2_ deduced from its high resolution electrospray ionization mass spectroscopy (HRESIMS) ions at *m*/*z* [M + H]^+^ 371.1499 (calcd. for C_22_H_19_N_4_O_2_, 371.1508) and [M + Na]^+^ 393.1322 (calcd. for C_22_H_18_N_4_NaO_2_, 393.1327). Extensive nuclear magnetic resonance (NMR) spectroscopic interpretation indicated that compound **1** is composed of three parts of indole (A), pyrazines (B), and 3-hydroxy-2-indolinon (C) through connection of two methylenes. The presence of the indolyl group was suggested by its NMR signals at *δ*_C_ 123.4 (CH, C-1), 111.6 (C, C-2), 126.8 (C, C-3), 118.4 (CH, C-4), 118.4 (CH, C-5), 121.0 (CH, C-6), 111.4 (CH, C-7), 136.3 (C, C-8), and *δ*_H_ 10.90 (1H, s, NH-1), 7.06 (1H, d, 1.8 Hz, H-1), 7.34 (1H, d, 8.0 Hz, H-4), 6.91 (1H, t, 8.0 Hz, H-5), 7.03 (1H, t, 8.0 Hz, H-6), and 7.32 (1H, d, 8.0 Hz, H-7) (Table 1). Similarly, NMR signals at *δ*_C_ 154.0 (C, C-10), 142.6 (CH, C-11), 144.4 (CH, C-12), 148.7 (C, C-13), and *δ*_H_ 8.28 (1H, d, 2.5 Hz, H-11) and 8.25 (1H, d, 2.5 Hz, H-12) were assigned to the pyrazine unit. The 3-hydroxy-2-indolinon moiety resonated at *δ*_C_ 75.4 (C, C-15), 178.3 (C, C-16), 141.5 (C, C-17), 109.3 (CH, C-18), 128.9 (CH, C-19), 121.2 (CH, C-20), 124.6 (CH, C-21), 130.6 (C, C-22), and *δ*_H_ 6.24 (1H, s, OH-15), 10.17 (1H, s, NH-16), 6.63 (1H, d, 7.8 Hz, H-18), 7.08 (1H, ddd, 7.8 and 1.1 Hz, H-19), 6.80 (1H, t, 7.8 Hz, H-20), and 6.90 (1H, d, 7.8 Hz, H-21). In addition, the presence of the two methylenes was confirmed by their NMR signals at *δ*_C_ 30.9 (CH_2_, C-9) and 42.6 (CH_2_, C-14) as well as *δ*_H_ 4.11 (2H, s, H-9), 3.29 (1H, d, 13.2 Hz, H-14a), and 3.12 (1H, d, 13.2 Hz, H-14b). As depicted in Figure 2, heteronuclear multiple bond coherence (HMBC) correlations of H-9 with C-1, C-2, C-3, C-10, and C-11 established that the indolyl group was linked to the pyrazine unit via a methylene. In the same way, HMBC correlations of H-14 with C-12, C-13, C-15, C-16, and C-22 confirmed the connection of the pyrazine unit and the 3-hydroxy-2-indolinon moiety. The absolute configuration of **1** at C-15 was determined based on the result of electronic circular dichroism (ECD) calculation. As shown in Figure 3, the experimental ECD spectrum of **1** showed a good agreement with the calculated ECD curve of *S* of **1**. Thus, the absolute configuration of **1** was assigned as 15*S*. Based on the foregoing evidence, the structure of **1** was elucidated as a new alkaloid, named indolepyrazine A. Its ^13^C and ^1^H NMR data (Table 1) are fully assigned based on heteronuclear singular quantum correlation (HSQC), ^1^H-^1^H correlated spectroscopy (^1^H-^1^H COSY), and HMBC correlations (Figure 2). To the best of our knowledge, natural alkaloids embodying an indole-pyrazine-oxindole skeleton are very rare and indolepyrazine A is the first report of this type of alkaloids.

The molecular formula C_14_H_13_N_3_ for compound **2** was deduced from its HRESIMS ions at *m*/*z* [M + H]^+^ 224.1188 (calcd. for C_14_H_14_N_3_, 224.1188) and [M + Na]^+^ 246.1006 (calcd. for C_14_H_13_N_3_Na, 246.1007). Just like **1**, the ^13^C and ^1^H NMR spectra of **2** also showed characteristic signals for an indolyl group and a pyrazine unit. The indolyl group resonated at *δ*_C_ 123.6 (CH, C-1), 111.5 (C, C-2), 126.9 (C, C-3), 118.5 (CH, C-4), 118.5 (CH, C-5), 121.1 (CH, C-6), 111.5 (CH, C-7), 136.3 (C, C-8) and *δ*_H_ 10.92 (1H, s, NH-1), 7.21 (1H, d, 2.3 Hz, H-1), 7.47 (1H, d, 8.0 Hz, H-4), 6.93 (1H, t, 8.0 Hz, H-5), 7.04 (1H, ddd, 8.0 and 1.0 Hz, H-6), and 7.33 (1H, d, 8.0 Hz, H-7). The NMR signals at *δ*_C_ 155.6 (C, C-10), 141.1 (CH, C-11), 141.6 (CH, C-12), 152.5 (C, C-13), and *δ*_H_ 8.35 (1H, s, H-11) and 8.32 (1H, s, H-12) were assigned to the pyrazine unit. In addition, the NMR spectra of **2** also showed the presence of a methylene (*δ*_C_ 31.3, CH_2_, C-9; *δ*_H_ 4.17, 2H, s, H-9) and a methyl (*δ*_C_ 21.1, CH_3_, C-14; *δ*_H_ 2.46, 3H, s, H-14). HMBC correlations (Figure 2) of H-9 with C-1, C-2, C-3, C-10, and C-11 established the linkage of the indolyl group and the pyrazine unit. The methyl group at C-13 was confirmed by the following HMBC correlations: H_3_-14 with C-12 and C-13, H-11 with C-10 and C-12, and H-12 with C-11 and C-13. Thus, the structure of **2** was determined as a new alkaloid, named indolepyrazine B. The assignment of its ^13^C and ^1^H NMR data (Table 1) was made by HSQC, ^1^H-^1^H COSY, and HMBC correlations (Figure 2).

The antimicrobial activities of the isolated compounds **1** and **2** against MRSA, *E*. *coli*, and *C. albicans* were determined by the microbroth dilution method [15]. Gentamicin (an antibiotic against both Gram-positive and Gram-negative bacteria) and amphotericin B (an antifungal drug) were used as positive controls. The results (Table 2) indicated that both **1** and **2** have antimicrobial activities in inhibiting the growth of MRSA (MIC: 12 μg/mL), *E. coli* (MIC: 8–10 μg/mL), and *C. albicans* (12–14 μg/mL). Positive control gentamicin had antibacterial activity with an MIC value of 3 μg/mL against MRSA or 0.5 μg/mL against *E. coli*; while amphotericin B gave an MIC value of 3 μg/mL against *C. albicans*.

## 3. Materials and Methods

### 3.1. General Experimental Procedures

HRESIMS data were acquired on an Agilent 6230 time of flight liquid chromatography/mass spectrometry (TOF LC/MS) spectrometer. NMR spectra were recorded on a JEOL 600 spectrometer using standard pulse programs and acquisition parameters. Chemical shifts were presented in *δ* (ppm) and referred to the NMR solvent used. ECD were measured on a JASCO J-815 spectropolarimeter (JASCO Corporation, Tokyo, Japan). Optical rotation and UV spectra were obtained on a RUDOLPH AutopolⅠAutomatic polarimeter and METASH UV-8000 (Shanghai METASH Instruments Co. Ltd., China), respectively. Octadecyl-functionalized silica gel (ODS, Cosmosil 75C_18_-Prep, Nacalai Tesque Inc., Kyoto, Japan) and sephadex LH-20 (GE Healthcare Bio-Sciences AB, Uppsala, Sweden) were used for column chromatography. HPLC separation was performed on a CXTH LC-3000 preparative HPLC system (Beijing Chuangxintongheng Science & Technology Co. Ltd., China). All solvents used were purchased from the Sinopharm Chemical Reagent Co. Ltd. (Shanghai, China). Methicillin-resistant *Staphylococcus aureus* (MRSA) ATCC 43300, *Escherichia coli* ATCC 25922, and *Candida albicans* were provided by Drs. Zhongjun Ma, Pinmei Wang, and Bin Wu, respectively. Amphotericin B (>95.0%) and gentamicin (99.6%) were ordered from Meilune Biotechnology Co. Ltd. (Dalian, China). Different culture media were made in the authors′ laboratory, including B liquid medium (soluble starch 20 g, KNO_3_ 1 g, K_2_HPO_4_ 0.5 g, MgSO_4_⋅7H_2_O 0.5 g, NaCl 0.5 g, FeSO_4_ 0.01 g, water 1 L), BY liquid medium (soluble starch 20 g, KNO_3_ 1g, K_2_HPO_4_ 0.5 g, MgSO_4_⋅7H_2_O 0.5 g, NaCl 0.5 g, FeSO_4_ 0.01 g, sea salt 35 g, water 1 L), D liquid medium (potatoes 200 g of 1 cm^3^ cubes, glucose 20 g, boiled into 1 L of water for 15 min), DY liquid medium (potatoes 200 g of 1 cm^3^ cubes, glucose 20 g, sea salt 35 g, boiled into 1 L of water for 15 min), E liquid medium (yeast 1.0 g, tryptone 5.0 g, FeCl_3_⋅6H_2_O 0.17 g, KH_2_PO_4_ 0.12 g, water 1 L), and EY liquid medium (yeast 1.0 g, tryptone 5.0 g, FeCl_3_⋅6H_2_O 0.17 g, KH_2_PO_4_ 0.12 g, sea salt 35 g, water 1 L).

### 3.2. Isolation and Identification of Strain ZZ1275

The strain ZZ1275 was isolated from a mud sample, which was collected from the coastal area of Karachi, Sindh, Pakistan during September 2017. The mud sample (1.0 g) was suspended into 10 mL of sterile water in a test tube and the mixture was stirred on a shaker for 30 min in order to release microorganisms bound to the mud particles. The sample (10^−1^ g/mL) was serially diluted to be 10^−2^ to 10^−4^ g/mL and 100 μL of each dilution was transferred into six different media of B, BY, D, DY, E, and EY in plates and then incubated at room temperature for one month. The single pure colony of strain ZZ1275 in E medium from the 10^–4^ g/mL dilution was picked with sterile needle and then transferred to another E medium plate. After another six days of incubation at 28 °C, the single colony of strain ZZ1275 that grew well was transferred onto E medium slant and then stored at 4 °C for further experiment.

The strain ZZ1275 was identified by 16S rDNA sequence analysis, which was conducted by Legenomics (Hangzhou, China), and its top DNA sequence using BLAST (nucleotide Basic Local Alignment Search Tool) was compared to the GenBank database. The result of the 16S rDNA sequence of strain ZZ1275 has been deposited in GenBank with an accession number MH746822. The voucher strain of *Acinetobacter* sp. ZZ1275 was conserved at the Laboratory of Institute of Marine Biology and Pharmacology, Ocean College, Zhoushan campus, Zhejiang University, Zhoushan, China.

### 3.3. Large Scale Culture of Strain ZZ1275

Briefly, the pure colony of strain ZZ1275 from the E medium slant was refreshed on the plate of E solid medium at 28 °C for 4 days. The pure colony of strain ZZ1275 was transferred into eight Erlenmeyer flasks (500 mL), each containing 250 mL of E liquid medium, and then incubated at 28 °C for 3 days on a rotary shaker at 180 rpm to prepare seed broth of strain ZZ1275. A seed broth of 5 mL from strain ZZ1275 was inoculated into 250 mL E medium in 500 mL Erlenmeyer flasks and then incubated at 28 °C for 12 days on a rotary shaker at 180 rpm. A total of 65 liters of E medium was prepared for this study.

### 3.4. Isolation of Indolepyrazines A (**1**) and B (**2**)

The scale up culture of strain ZZ1275 in E medium was centrifuged to separate pellets from broth. The pellets and broth were respectively extracted by MeOH and EtOAc three times. A combination of the MeOH and EtOAc extract was fractionated on a column of ODS (100 g) eluting with 30%, 50%, 60%, 70%, 80%, and 100% MeOH to afford six fractions of 30M, 50M, 60M, 70M, 80M, and 100M. Fraction 60M was further separated on a sephadex LH-20 column eluting with 70% MeOH to give 20 fractions (each 10 mL). The combined fractions (Frs. 14–20) were further separated by HPLC using a Fuji C_18_ column (250 × 30 mm, 10 µm) with isocratic mobile phase of 58% MeOH in water and flow rate of 10 mL/min to afford compound **1** (10 mg, t_R_ 32.5 min) and compound **2** (3 mg, t_R_ 45.2 min).

Indolepyrazine A (**1**): yellow amorphous powder; molecular formula C_22_H_18_N_4_O_2_; ${[\alpha ]\;}_{D}^{20}$ +11.5° (*c* 0.1, MeOH); ECD (10 mg/L, MeOH) λmax (∆ε) 205 (−12.54), 208 (−13.67), 241 (+11.25), 265 (−2.67), 278 (+0.87), 296 (−2.44) nm; UV (MeOH) λmax (log ε) 250 (3.41), 272 (3.51) nm; ^13^C and ^1^H NMR data (in DMSO-*d*_6_), see Table 1; HRESIMS *m*/*z* [M + H]^+^ 371.1499 (calcd. for C_22_H_19_N_4_O_2_, 371.1508) and [M + Na]^+^ 393.1322 (calcd. for C_22_H_18_N_4_NaO_2_, 393.1327).

Indolepyrazine B (**2**): colorless solid; molecular formula C_14_H_13_N_3_; UV (MeOH) λmax (log ε) 222 (3.99), 275 (3.54) nm; ^13^C and ^1^H NMR data (in DMSO-*d*_6_), see Table 1; HRESIMS *m*/*z* [M + H]^+^ 224.1188 (calcd. for C_14_H_14_N_3_, 224.1188) and [M + Na]^+^ 246.1006 (calcd. for C_14_H_13_N_3_Na, 246.1007).

### 3.5. ECD Calculation

Monte Carlo conformational searches were carried out by means of the Spartan′s 10 software using Merck Molecular Force Field (MMFF). The conformers with Boltzmann-population of over 5% were chosen for ECD calculations, and then the conformers were initially optimized at B3LYP/6-31+g (d, p) level in MeOH using the conductor-like polarizable continuum model (CPCM) (Table S2). The theoretical calculation of ECD was conducted in MeOH using the Time-dependent Density functional theory (TD-DFT) at the B3LYP/6-311+g (d, p) level for all conformers of compound **1** (Table S3). Rotatory strengths for a total of 50 excited states were calculated. ECD spectra were generated using the program SpecDis 1.6 (University of Wurzburg, Wurzburg, Germany) and GraphPad Prism 5 (University of California San Diego, USA) from dipole-length rotational strengths by applying Gaussian band shapes with sigma = 0.3 eV. To get the final spectra, the simulated spectra of the conformers were averaged according to the Boltzmann distribution theory and their relative Gibbs free energy (ΔG). Theoretical ECD spectrum of the corresponding enantiomer were obtained through direct inverse of the ECD spectrum of the above-mentioned compounds. By comparing the experimental spectrum with the calculated ECD spectra, the chiral center of **1** was determined.

### 3.6. Antimicrobial Activive Assay

The antimicrobial activities of indolepyrazines A (**1**) and B (**2**) against MRSA, *E. coli*, and *C. albicans* were evaluated by the microbroth dilution method as described in the previous study [15].

## 4. Conclusions

Secondary metabolites from marine microorganisms are important sources for the discovery of novel bioactive natural products. In this study, a Gram-negative bacterial strain *Acinetobacter* sp. ZZ1725 was isolated from a coastal mud sample. Two previously undescribed alkaloids indolepyrazines A and B were isolated and identified from a scale-up culture of strain ZZ1275 in the E medium. Indolepyrazine A is a very rare alkaloid with an indole-pyrazine-oxindole skeleton and represents the first example of this type of compounds. Indolepyrazines A and B also are the first reports of secondary metabolites from marine-sourced *Acinetobacter* species. Both compounds showed inhibitory activities against the growth of MRSA, *E*. *coli*, and *C. albicans*, which might be responsible for the antimicrobial activities of the crude extract.

## Figures and Tables

**Table 1:** ^13^C (150 MHz) and ^1^H (600 MHz) nuclear magnetic resonance (NMR) data of indolepyrazines A (**1**) and B (**2**) (in DMSO-*d*_6_).

1						2		
No.	^13^C, Type	^1^H (*J* in Hz)	No.	^13^C, Type	^1^H (*J* in Hz)	No.	^13^C, Type	^1^H (*J* in Hz)
1	123.4, CH	7.06, d (1.8)	16	178.3, C	–	1	123.6, CH	7.21, d (2.3)
2	111.6, C	–	17	141.5, C	–	2	111.5, C	–
3	126.8, C	–	18	109.3, CH	6.63, d (7.8)	3	126.9, C	–
4	118.4, CH	7.34, d (8.0)	19	128.9, CH	7.08, ddd (7.8, 1.1)	4	118.5, CH	7.47, d (8.0)
5	118.4, CH	6.91, t (8.0)	20	121.2, CH	6.80, t (7.8)	5	118.5, CH	6.93, t (8.0)
6	121.0, CH	7.04, t (8.0)	21	124.6, CH	6.90, d (7.8)	6	121.1, CH	7.04, ddd (8.0, 1.0)
7	111.4, CH	7.32, d (8.0)	22	130.6, C	–	7	111.5, CH	7.33, d (8.0)
8	136.3, C	–	NH-1	–	10.90, s	8	136.3, C	–
9	30.9, CH_2_	4.11, s	NH-16	–	10.17, s	9	31.3, CH_2_	4.17, s
10	154.0, C	–	OH-15	–	6.24, s	10	155.6, C	–
11	142.6, CH	8.28, d (2.5)				11	141.1, CH	8.35, s
12	144.4, CH	8.25, d (2.5)				12	141.6, CH	8.32, s
13	148.7, C	–				13	152.5, C	–
14	42.5, CH_2_	3.12, d (13.2); 3.29, d (13.2)				14	21.1, CH_3_	2.46, s
15	75.4, C	–				NH-1	–	10.92, s

**Table 2:** Antimicrobial activities of indolepyrazines A (**1**) and B (**2**) (MIC in μg/mL).

Microorganisms	1	2	Gentamicin	Amphotericin B
MRSA	12	12	3	–
*E. coli*	10	8	0.5	–
*C. albicans*	12	14	–	3

## Supplementary Materials

The following materials are available online at http://www.mdpi.com/1660-3397/17/2/89/s1. Figure S1: 16S rDNA sequence of *Acinetobacter* sp. ZZ1275; Figure S2: Colony picture of strain ZZ1275; Figures S3–S17: NMR, HRESIMS, and UV spectra of indolepyrazine A (**1**); Figures S18–S29: NMR, HRESIMS, and UV spectra of indolepyrazine B (**2**); Table S1: Sequences producing significant alignments of strain ZZ1275; Table S2: Gibbs free energies and equilibrium population of low-energy conformers (**1a**–**1d**) of indolepyrazine A (**1**); and Table S3: Cartesian coordinates for the low-energy reoptimized MMFF conformers of indolepyrazine A (**1**) at B3LYP/6-311+G(d,p) level of theory in MeOH.
