# Supplementary material for: Novel Antimicrobial Indolepyrazines A and B from the Marine-Associated Acinetobacter sp. ZZ1275
Source: Mar Drugs. 2019 Feb 1;17(2):89. doi: 10.3390/md17020089 (PMC6410138; doi:10.3390/md17020089)

Supplementary Materials for

Novel Antimicrobial Indolepyrazines A and B from the Marine-Associated *Acinetobacter* sp. ZZ1275

Komal Anjum [1], Sidra Kaleem [2], Wenwen Yi [2], Guowan Zheng [1], Xiaoyuan Lian [1,*] and Zhizhen Zhang [2,*]

[1] College of Pharmaceutical Sciences, Zhejiang University, Hangzhou 310058, China; komalazam@ymail.com (K.A.), 0818331@163.com (G.Z.)

[2] Ocean College, Zhoushan Campus, Zhejiang University, Zhoushan 316021, China; kaleemsidra85@yahoo.com (S.K.), 18790658213@163.com (W.Y.)

* Correspondence: xylian@zju.edu.cn (X.L.); zzhang88@zju.edu.cn (Z.Z.); Tel.: +86-135-7547-6388 (X.L.); +86-136-7585-9706 (Z.Z.)

CONTENT

Figure S_1. 16S rDNA sequence of strain ZZ1275……………………………………..3

Figure S_2. Colony picture of strain ZZ1275……………………………….…..3

Table S_1. Sequences producing significant alignments of strain ZZ1275……………..4

Figures S_{3-5}. ^{1}H NMR spectra of indolepyrazine A (**1**)……………………….…..…....4

Figures S_{6-9}. ^{13}C NMR spectra of indolepyrazine A (**1**)…..……………………………6

Figure S_{10}. ^{1}H-^{1}H COSY spectrum of indolepyrazine A (**1**)…………………………...8

Figures S_{11-12}. HSQC spectra of indolepyrazine A (**1**)……….………………………..8

Figures S_{13-15}. HMBC spectra of indolepyrazine A (**1**)…..…………………………......9

Figure S_{16}. HRESIMS spectrum of indolepyrazine A (**1**)……………………….…11

Figure S_{17}. UV spectrum of indolepyrazine A (**1**)…………….………………………11

Table S_2. Gibbs free energies^{a} and equilibrium populations^{b} of low-energy conformers (**1a**–**1d**) of indolepyrazine A (**1**)…………………………………………………...12

Table S_3. Cartesian coordinates for the low-energy reoptimized MMFF conformers of indolepyrazine A (**1**) at B3LYP/6-311+G(d,p) level of theory in MeOH…..………..….12

Figures S_{18-20}. ^{1}H NMR spectra of indolepyrazine B (**2**)……….……………………17

Figures S_{21-22}. ^{13}C NMR spectra of indolepyrazine B (**2**)…..…………………………...18

Figure S_{23}. ^{1}H-^{1}H COSY spectrum of indolepyrazine B (**2**)…………….………….19

Figures S_{24-25}. HSQC spectra of indolepyrazine B (**2**)……………………...………20

Figures S_{26-27}. HMBC spectra of indolepyrazine B (**2**)…………………...………...21

Figure S_{28}. HRESIMS spectrum of indolepyrazine B (**2**)……………………………22

Figure S_{29}. UV spectrum of indolepyrazine B (**2**)……………….……………….………22

Figure S_1. 16S rDNA sequence of strain ZZ1275

GCGTTGGTACCGCCCTCTTTGCAGTTATGCTACTACTTCTGGTGCAGCAAACTCCC
ATGGTGAGACGGGCGGTGTGGACAAGGCCCGGGAACGTATTCTCCGCGGCATTCT
GATCCGCGATTACTAACGATTCCGACTTCATGGAGGCGAGTTGCAGACTCAAATCC
GGACTACGATCGACTTTTTGATATTAACATCCTATCGCTAGGTAGCACCCCTTTGTA
CCGACCATTGTATGACGTGTGTAGCCCTGGCCGTAAGGGCCATGATGACTTGACGT
CGTCCCCGCCTTCCTCCAGTTTGTCACTGCCCGGATCCTTAAAGTTCCCATCCGAA
ATGCTGGAAAGTAAGGAAAAGGGTTGCGCTCGTTGCGGGACTTAACCCAACATCT
CACGACCCGAGCTGACGACAGCCATGCGACACCTGTATGTAGATTCCCGAAGGCA
CCAATCCATCTCTGGAAATCTCTACTATGTCAAGGCCAAGGAAGGTTCTTCGCGTT
GCATCGAATTAAACCACATGCTCCACCGCTTGTGCGGGCCCCCGTCAATTCATTTG
AGTTTTASTCTTGMGAYCGTACTMGYCAGGCGRTCTASTTATCGYGTTAGMTGCGG
CACTAAAGCTTCAAAGGCCCCAACGGGTAGTAGACATCGTTTACGGCATGGATTAC
CAGGGTATTTAATCCTGTTTGCTCCACATGCTTTAGTCCTCAGCGTCAGTGTTAGTT
CAGATGTCTGCCTTCGTCATGGGTATTAGTTCAGATCTCTACGCATTTCTTCGTTAC
ACCTGGAATTCTACCATCCTCTCCCACAGTATAGCCAATCAGTATTGAATGCAATTA
CCAAGTTAAGATCGGGGATTTCACATTGGACTTAATTGGCCGCCTACGCGCGCTTT
ACGCACAGTAAATCCGATTTACGCTTGCACCATATGTATTGCCGCGGCTGGTGGCA
CAGAGTTAGCCGGTGCTTATTGTGCGAGTATAGTCCACTCATCTTAGGTATTAACTA
TGTGAGCCTCCTCTTCGCTTAAAGTGGTGTACAACCATAAGACGTTCTTCATACATG
CGGCATGGTTGGATCAGGGTTCTCTCCATTGTTCAATATTATTCACTGCTGCCTCCT
GTAGGAGTATGGTCCGTGTCTCAGTAGCAGTGTGGCGGATCATCGTCTCAGAAGCG
CTACAGATCGTCGCCTTGGTAGGCCTTTATTCCATCAACTAGATAATGAGAGTTAGG
GTCATCTATTAGCGCAAGGTCACAAGTGATCCCTTGCTTTCTCCCGTAGGACGTATG
CGGTATTAGCATCCCTTTCGAGATGTTGTCGTCCAATAATAGGCAGATTCCTAAGCA
TTACTCACTCGTCCGCCGGTAAGTGATAGTGCAAGCAACATGCATCTATCGCTCGA
CTTGCATGGTAGCCGCCCAGCC (1429 bp).

Figure S_2. Colony picture of strain ZZ1275

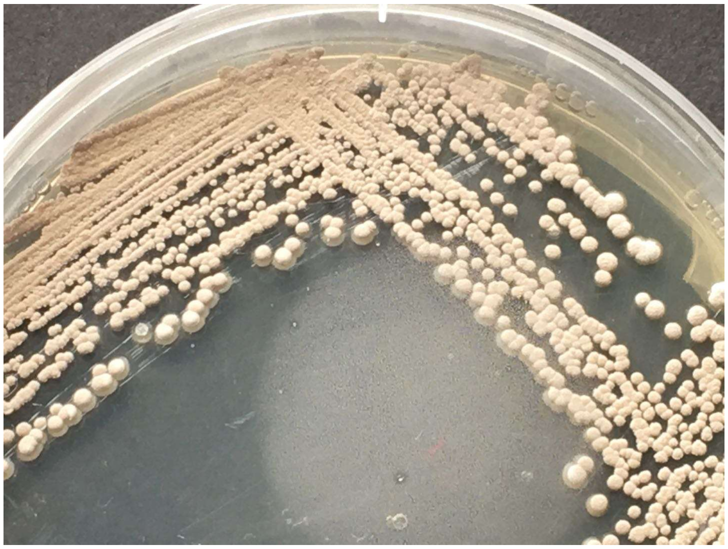

Table S_1. Sequences producing significant alignments of strain ZZ1275.

Accession	Description	Max score	Total score	Query coverage	Evalue	Ident
MH746822.1	*Acinetobacter baylyi* strain 16S ribosomal RNA gene, partial sequence	2606	2606	100%	0.0	100%
KY817316.1	*Acinetobacter baylyi* strain L7 16S ribosomal RNA gene, partial sequence	1969	1969	99%	0.0	92%
KU863622.1	Bacterium strain BPIC3 16S ribosomal RNA gene, partial sequence	1967	1967	98%	0.0	92%
MG011591.1	*Acinetobacter baylyi* strain MnW3201007 16S ribosomal RNA gene, partial sequence	1964	1964	98%	0.0	92%
KU922292.1	*Acinetobacter baumannii* strain L30 16 16S ribosomal RNA gene, partial sequence	1964	1964	98%	0.0	92%
KU922258.1	*Acinetobacter baumannii* strain L9 16S ribosomal RNA gene, partial sequence	1964	1964	98%	0.0	92%

Figure S_3. ^{1}H NMR spectrum of indolepyrazine A (**1**)

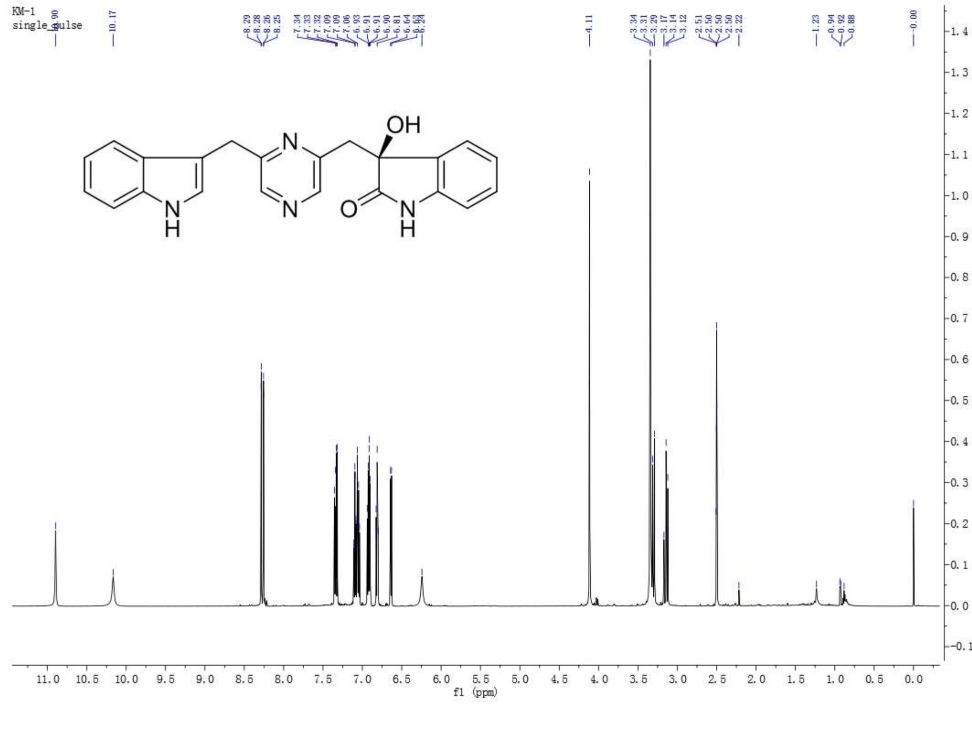

Figure S4. ^{1}H NMR spectrum of indolepyrazine A (**1**)

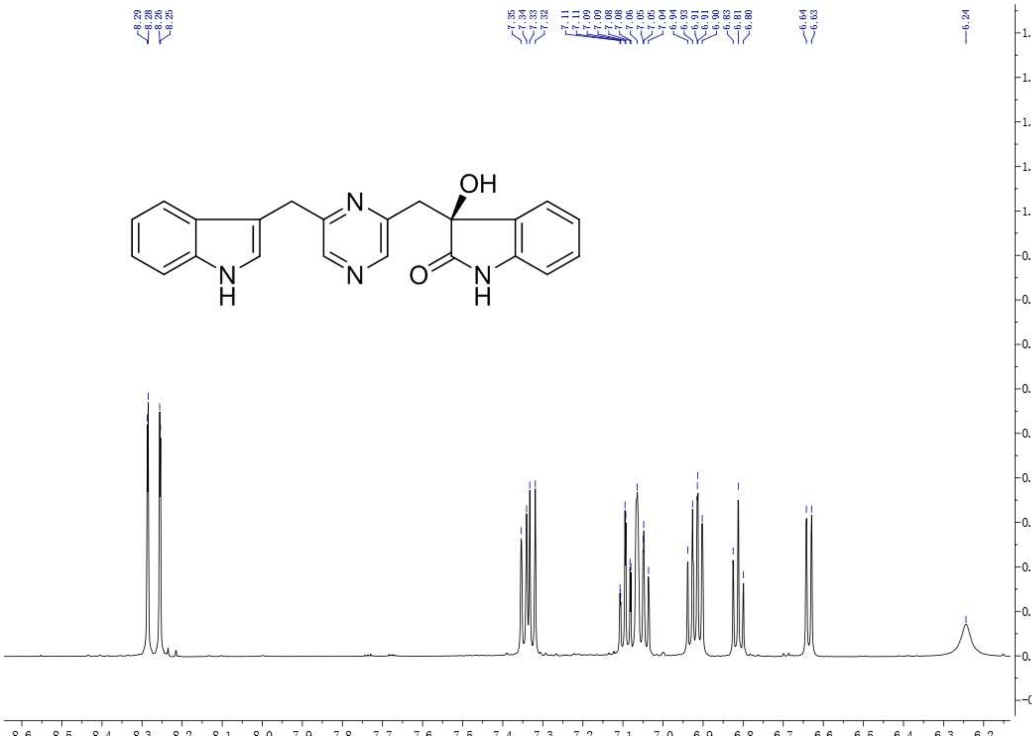

Figure S5. ^{1}H NMR spectrum of indolepyrazine A (**1**)

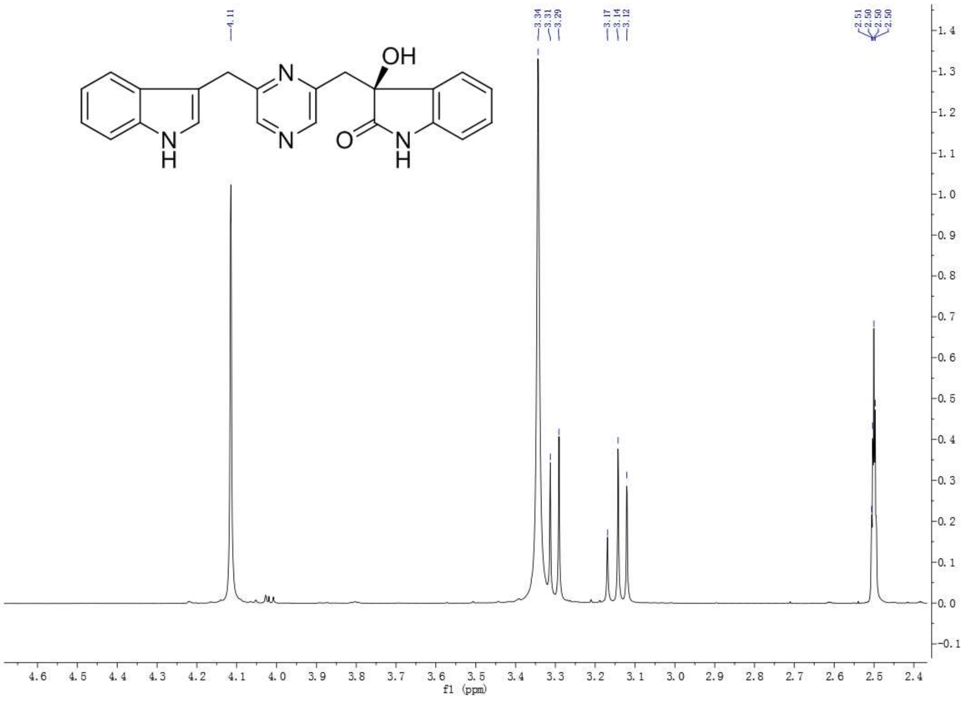

Figure S6. ^{13}C NMR spectrum of indolepyrazine A (**1**)

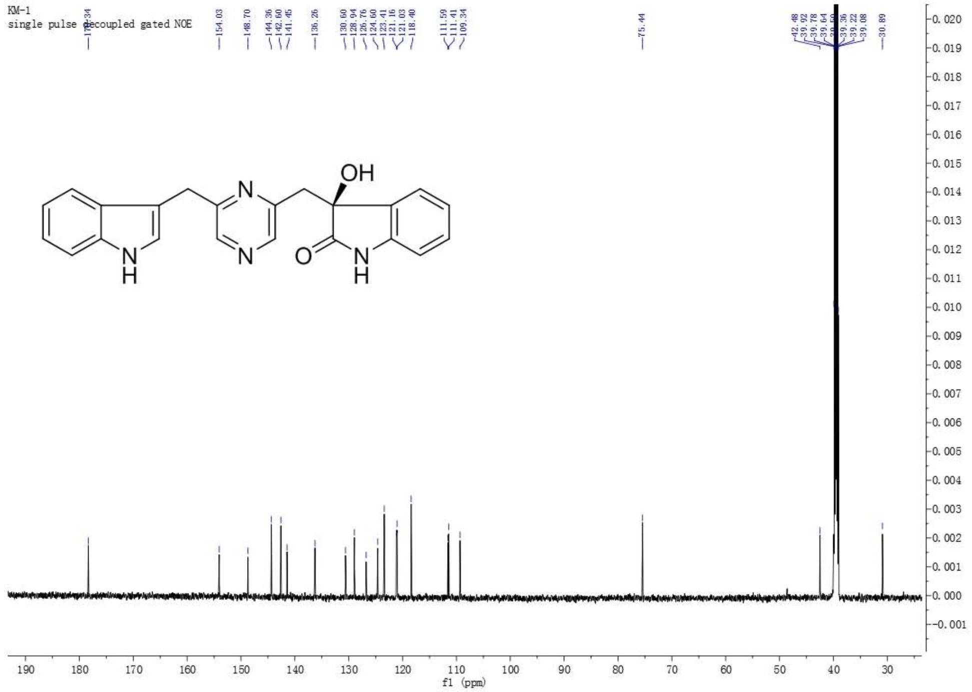

Figure S7. ^{13}C NMR spectrum of indolepyrazine A (**1**)

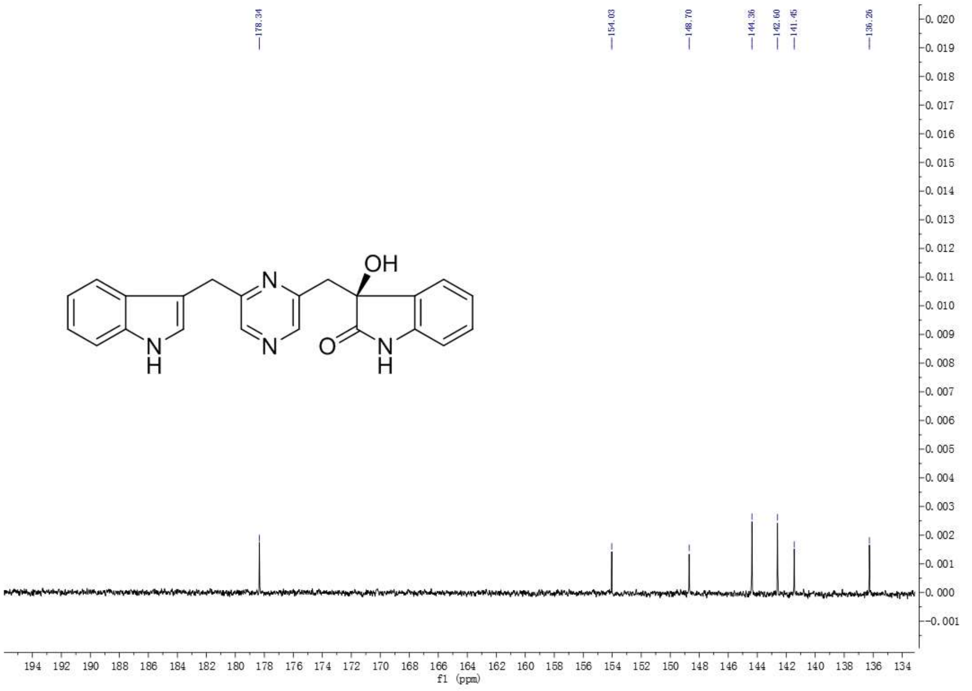

Figure S8. ^{13}C NMR spectrum of indolepyrazine A (**1**)

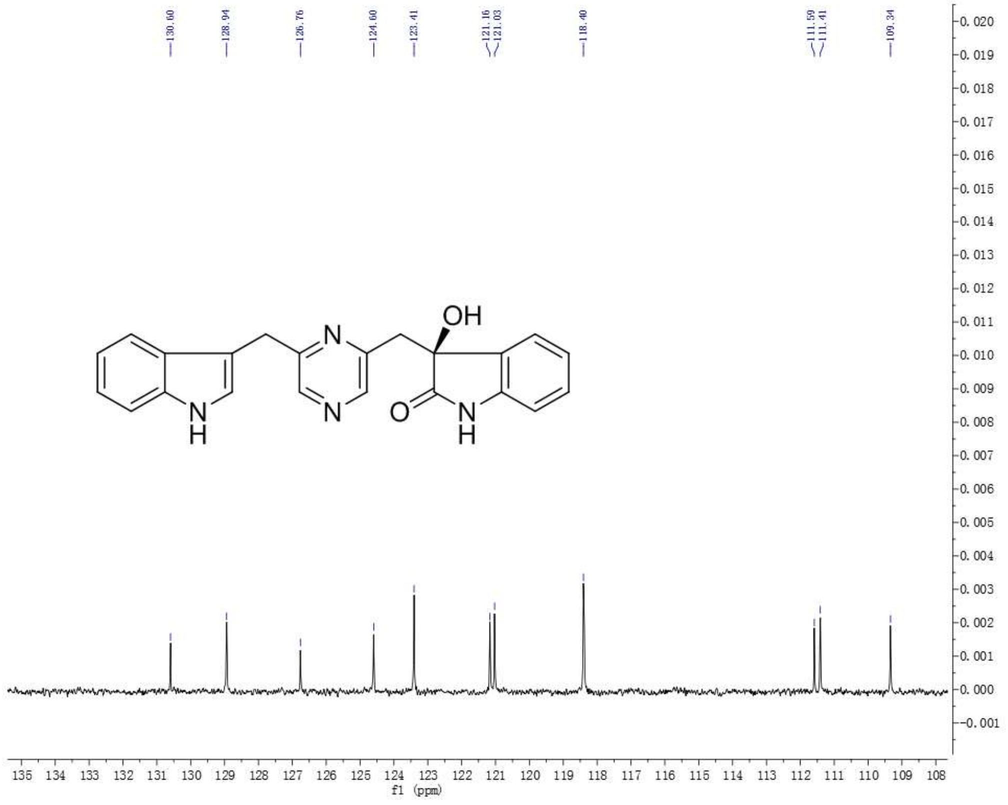

Figure S9. ^{13}C NMR spectrum of indolepyrazine A (**1**)

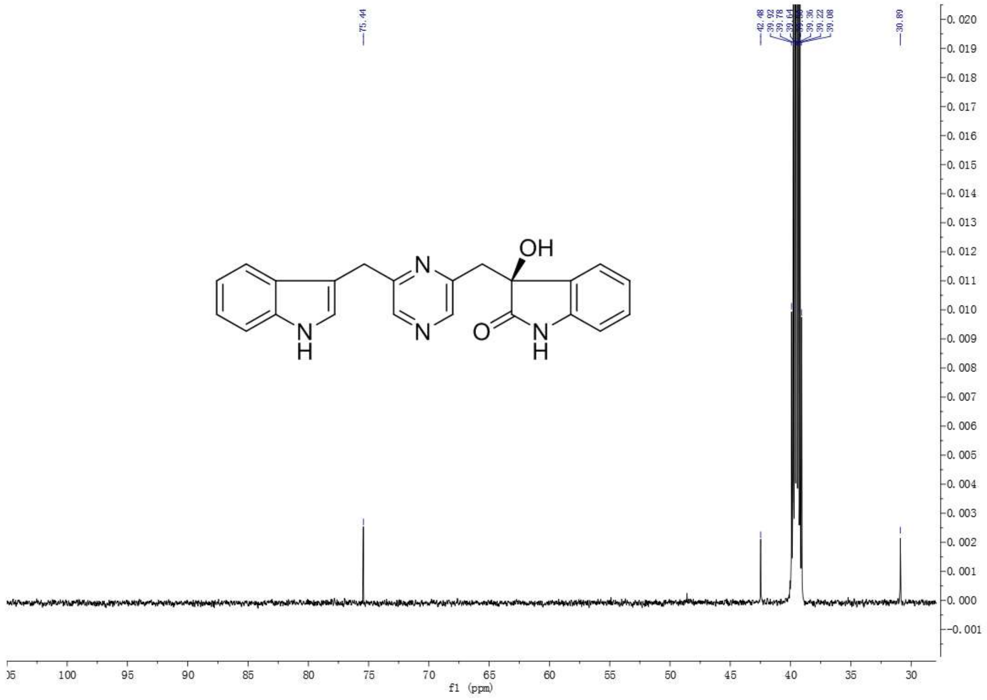

Figure S10. ^{1}H-^{1}H COSY spectrum of indolepyrazine A (**1**)

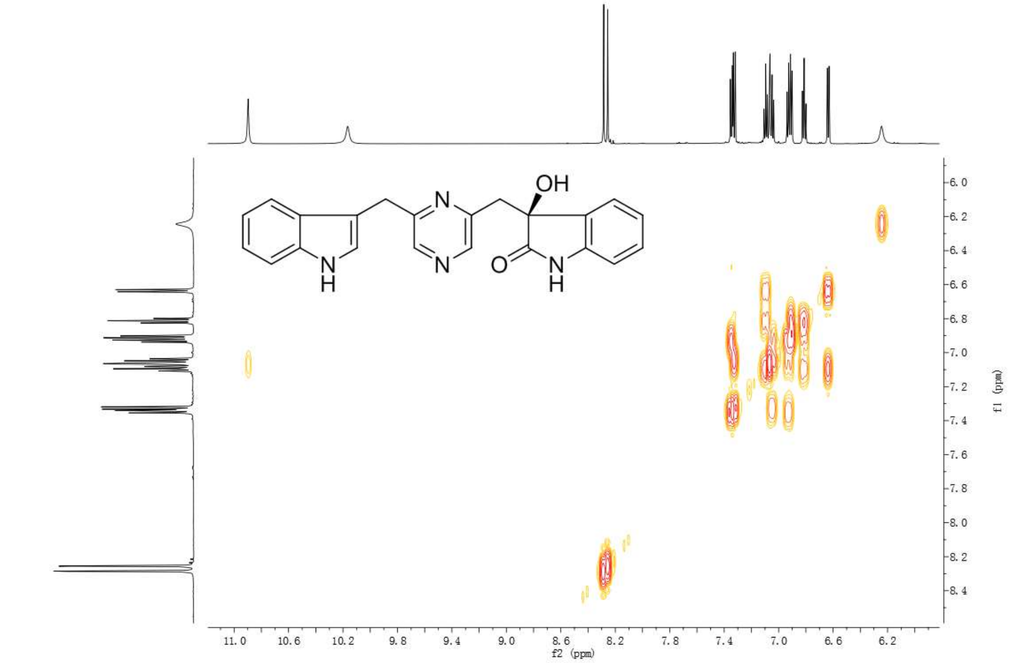

Figure S11. HSQC spectrum of indolepyrazine A (**1**)

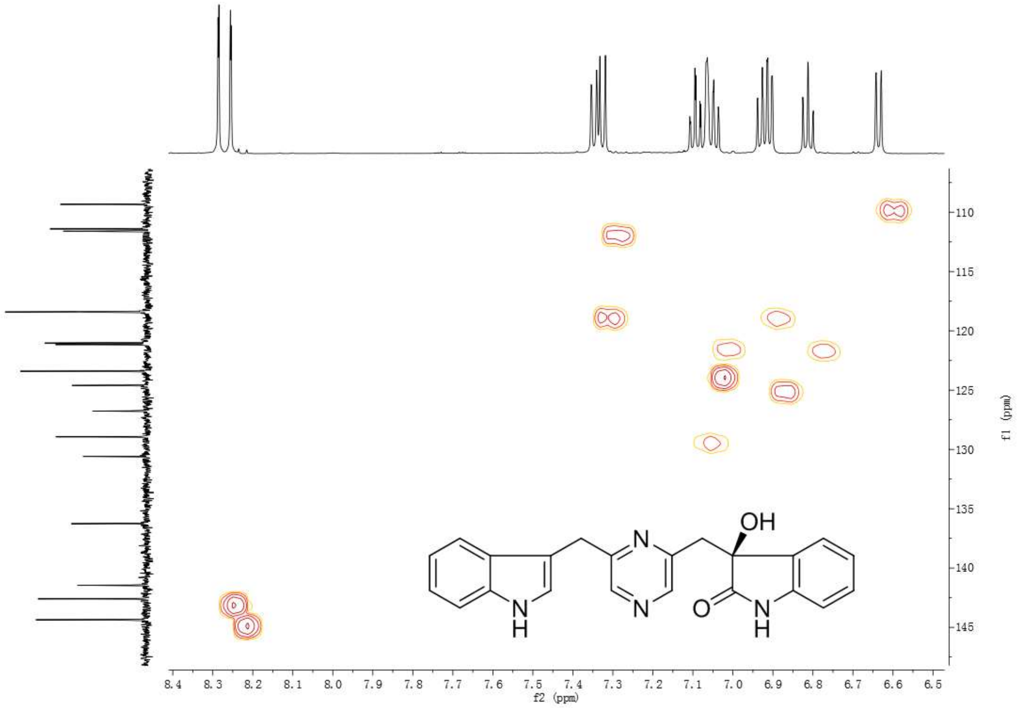

Figure S12. HSQC spectrum of indolepyrazine A (**1**)

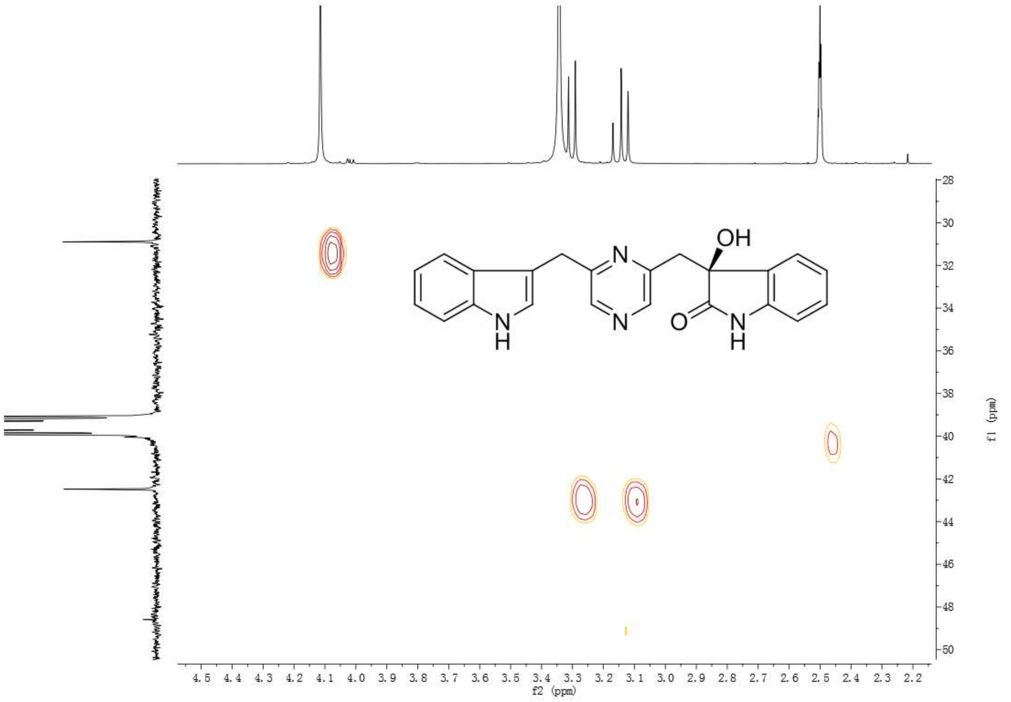

Figure S13. HMBC spectrum of indolepyrazine A (**1**)

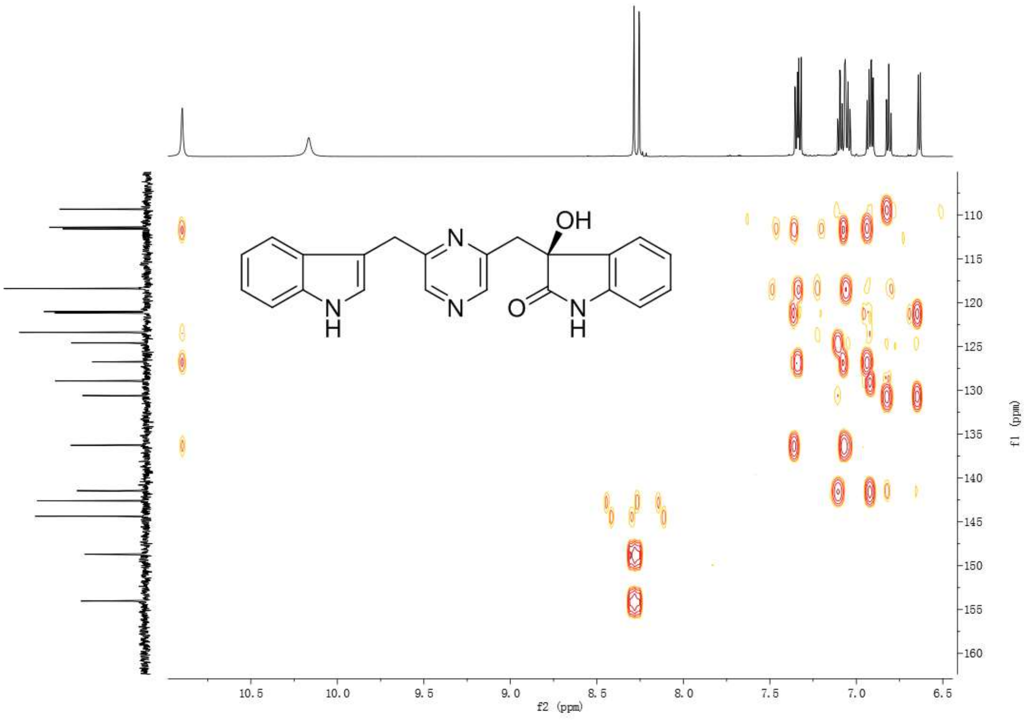

Figure S_{14}. HMBC spectrum of indolepyrazine A (**1**)

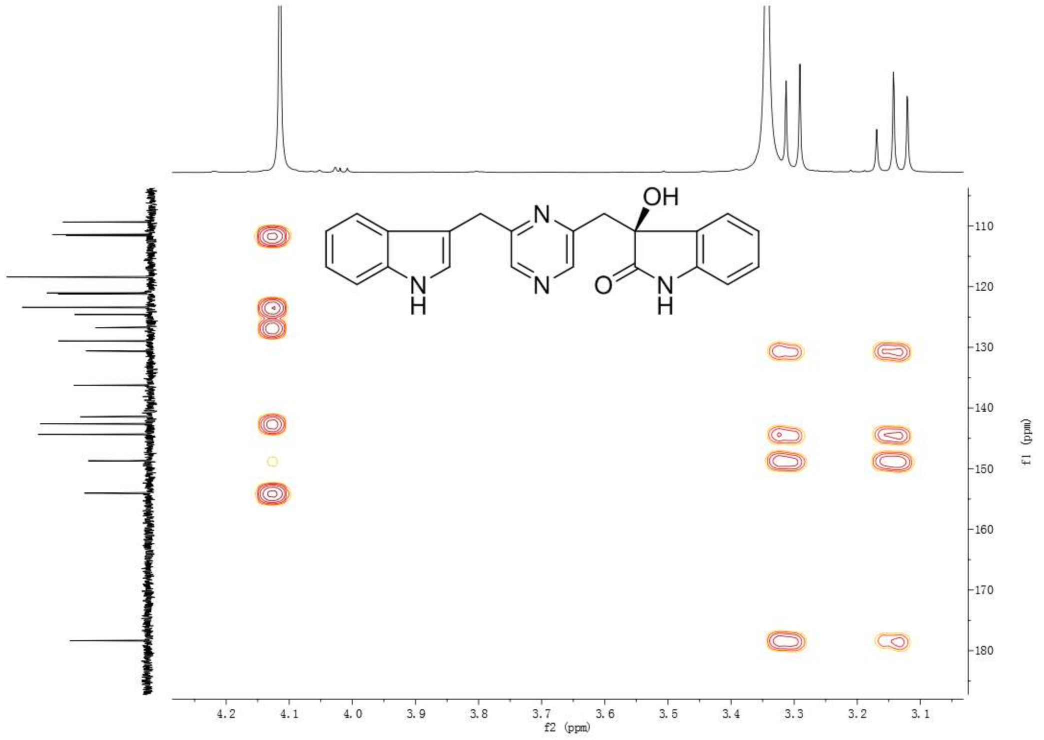

Figure S_{15}. HMBC spectrum of indolepyrazine A (**1**)

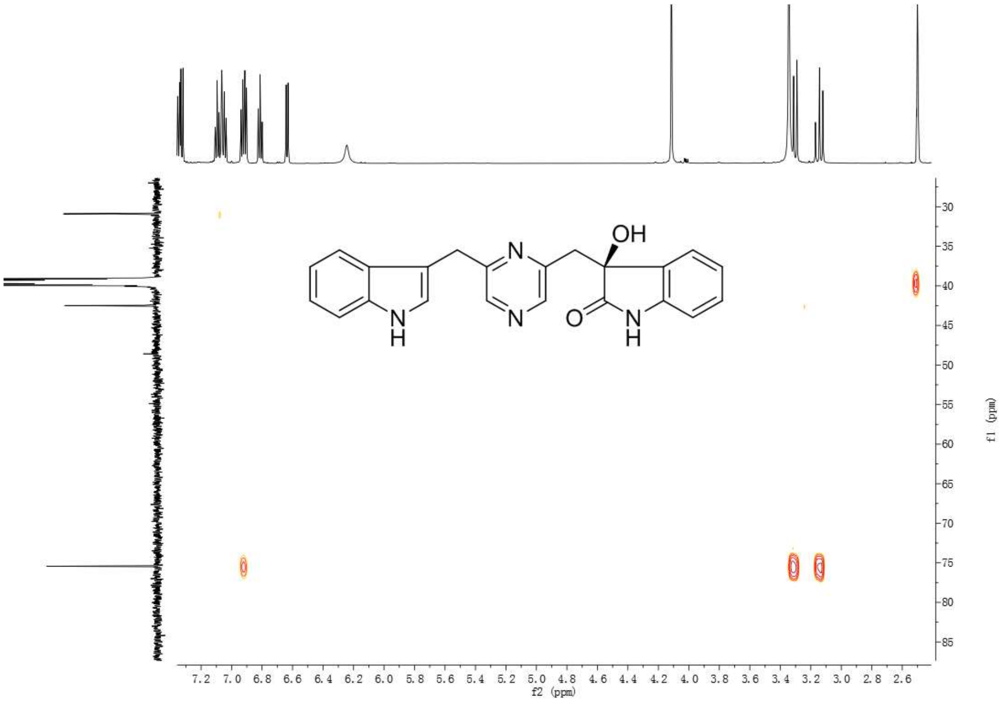

Figure S_{16}. HRESIMS spectrum of indolepyrazine A (**1**)

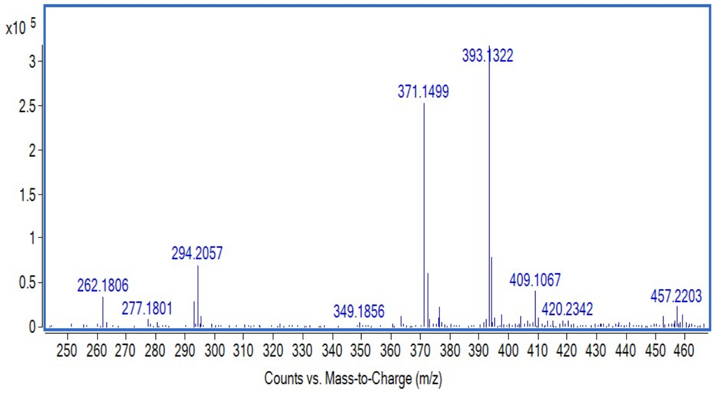

Figure S_{17}. UV spectrum of indolepyrazine A (**1**)

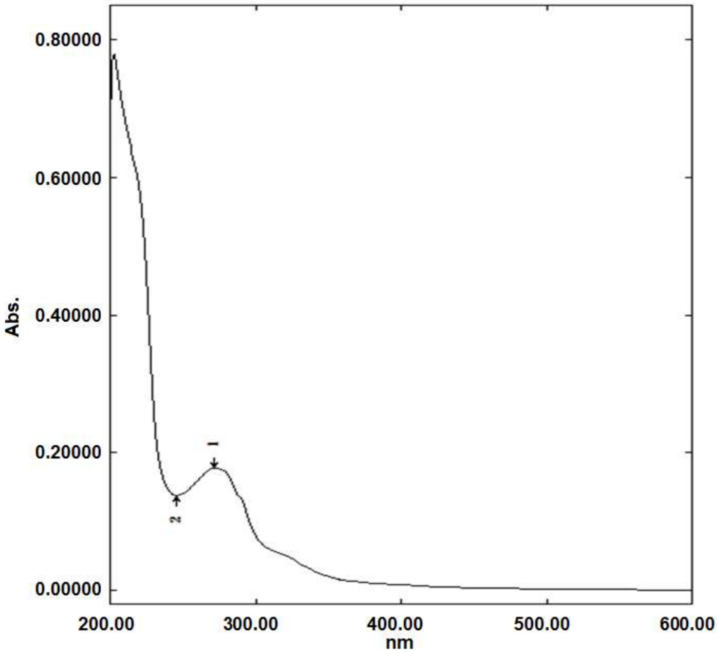

Table S2. Gibbs free energies and equilibrium populations of low-energy conformers (**1a**–**1d**) of indolepyrazine A (**1**).

Conformers	In MeOH	
	Δ*G*	Δ*G*
**1a**	0	0
**1b**	0.28	0.28
**1c**	1.78	1.78
**1d**	1.97	1.97

[a] B3LYP/6-31+G(d,p), in kcal/mol; [b] From Δ*G* values at 298.15K.

Table S3. Cartesian coordinates for the low-energy reoptimized MMFF conformers of indolepyrazine A (**1**) at B3LYP/6-311+G(d,p) level of theory in MeOH.

**1a**			Standard Orientation (Ångstroms)		
I	Atom	Type	X	Y	Z
1.	6.	0.	3.086124	1.549401	1.198165
2.	6.	0.	3.195205	1.779314	2.587679
3.	6.	0.	2.173161	2.400129	3.296731
4.	6.	0.	1.031211	2.785001	2.582869
5.	6.	0.	0.895102	2.557312	1.182855
6.	6.	0.	1.949102	1.930971	0.491902
7.	7.	0.	-0.117688	3.414669	3.007932
8.	6.	0.	-0.962281	3.595062	1.931690
9.	6.	0.	-0.389586	3.084611	0.790684
10.	6.	0.	-2.872760	1.343262	-0.507209
11.	6.	0.	-1.604149	1.727758	-0.967354
12.	6.	0.	-1.001366	3.068986	-0.587981
13.	7.	0.	-3.427826	0.172910	-0.830867
14.	6.	0.	-2.702500	-0.632961	-1.616304
15.	6.	0.	-1.431691	-0.276898	-2.082017
16.	7.	0.	-0.892799	0.911848	-1.756828
17.	6.	0.	1.502739	-1.158009	-1.538831
18.	6.	0.	0.441866	-2.062579	-2.227086
19.	6.	0.	-0.620703	-1.197741	-2.961462
20.	7.	0.	1.411779	-1.366620	-0.190820
21.	6.	0.	0.488449	-2.381987	0.119741
22.	6.	0.	-0.101016	-2.858817	-1.062014
23.	8.	0.	2.288595	-0.430146	-2.129540
24.	6.	0.	0.164357	-2.904245	1.365556
25.	6.	0.	-0.774117	-3.944533	1.404653
26.	6.	0.	-1.361800	-4.437899	0.237230
27.	6.	0.	-1.027407	-3.889878	-1.010273
28.	8.	0.	1.093417	-2.911345	-3.165207

29.	1.	0.	3.909041	1.074112	0.671332
30.	1.	0.	4.095194	1.469741	3.110584
31.	1.	0.	2.256637	2.581813	4.364068
32.	1.	0.	1.875825	1.737620	-0.573685
33.	1.	0.	-0.308417	3.715885	3.950987
34.	1.	0.	-1.917652	4.083810	2.062069
35.	1.	0.	-3.451808	2.007874	0.130199
36.	1.	0.	-0.247487	3.318116	-1.341603
37.	1.	0.	-1.781780	3.835631	-0.641712
38.	1.	0.	-3.144981	-1.587458	-1.889962
39.	1.	0.	-0.087304	-0.595811	-3.704907
40.	1.	0.	-1.277916	-1.892881	-3.491922
41.	1.	0.	1.974639	-0.860860	0.482347
42.	1.	0.	0.624413	-2.527996	2.273206
43.	1.	0.	-1.044134	-4.374397	2.364453
44.	1.	0.	-2.081374	-5.248078	0.295214
45.	1.	0.	-1.483543	-4.270313	-1.919452
46.	1.	0.	1.658231	-2.337383	-3.708111

**1b**	Standard Orientation (Ångstroms)				
I	Atom	Type	X	Y	Z
1.	6.	0.	2.181178	2.585657	-2.023817
2.	6.	0.	2.260933	3.848389	-2.648766
3.	6.	0.	1.600624	4.954006	-2.124100
4.	6.	0.	0.855527	4.766453	-0.953815
5.	6.	0.	0.759768	3.501368	-0.304357
6.	6.	0.	1.439762	2.401146	-0.862342
7.	7.	0.	0.112878	5.664052	-0.217200
8.	6.	0.	-0.439170	5.014883	0.866672
9.	6.	0.	-0.073400	3.689714	0.860523
10.	6.	0.	-2.819291	1.816112	1.292520
11.	6.	0.	-1.430887	1.617808	1.322951
12.	6.	0.	-0.479432	2.656208	1.881514
13.	7.	0.	-3.660427	0.908288	0.791210
14.	6.	0.	-3.116510	-0.210226	0.295721
15.	6.	0.	-1.735627	-0.428257	0.294248
16.	7.	0.	-0.908377	0.493864	0.818297
17.	6.	0.	-0.645410	-2.889403	1.875193
18.	6.	0.	-0.016398	-2.336359	0.551918
19.	6.	0.	-1.110392	-1.660379	-0.317948
20.	7.	0.	-0.528796	-4.256819	1.842324
21.	6.	0.	0.166388	-4.702798	0.705034
22.	6.	0.	0.511270	-3.601466	-0.092191
23.	8.	0.	-1.144669	-2.228343	2.772689
24.	6.	0.	0.510067	-6.001864	0.351863

25.	6.	0.	1.229639	-6.178587	-0.838241
26.	6.	0.	1.589365	-5.091109	-1.638054
27.	6.	0.	1.227808	-3.787933	-1.264322
28.	8.	0.	1.043811	-1.439921	0.838600
29.	1.	0.	2.708758	1.743512	-2.462342
30.	1.	0.	2.847766	3.959136	-3.555890
31.	1.	0.	1.660090	5.927110	-2.602430
32.	1.	0.	1.377574	1.421171	-0.399814
33.	1.	0.	0.001039	6.644351	-0.424305
34.	1.	0.	-1.054906	5.553521	1.573291
35.	1.	0.	-3.252419	2.730523	1.690866
36.	1.	0.	-0.954462	3.158788	2.729938
37.	1.	0.	0.401306	2.129022	2.265289
38.	1.	0.	-3.795220	-0.956115	-0.110306
39.	1.	0.	-0.627597	-1.376226	-1.261439
40.	1.	0.	-1.889174	-2.387609	-0.564984
41.	1.	0.	-0.832516	-4.849899	2.602763
42.	1.	0.	0.237995	-6.847867	0.974427
43.	1.	0.	1.513318	-7.182938	-1.138024
44.	1.	0.	2.151941	-5.254623	-2.551584
45.	1.	0.	1.510124	-2.938880	-1.879953
46.	1.	0.	0.616150	-0.578460	1.039376
**1c**			Standard Orientation (Ångstroms)		
I	Atom	Type	X	Y	Z
1.	6.	0.	-0.143689	1.765493	3.002700
2.	6.	0.	1.196029	2.087425	3.311638
3.	6.	0.	2.033904	2.672338	2.365935
4.	6.	0.	1.499704	2.925475	1.096031
5.	6.	0.	0.154876	2.595350	0.757421
6.	6.	0.	-0.667727	2.011406	1.738050
7.	7.	0.	2.078246	3.480314	-0.023719
8.	6.	0.	1.155459	3.495496	-1.050326
9.	6.	0.	-0.036579	2.963396	-0.624288
10.	6.	0.	-2.937171	0.826677	-1.586303
11.	6.	0.	-1.618947	1.292501	-1.682970
12.	6.	0.	-1.271294	2.755064	-1.455278
13.	7.	0.	-3.262858	-0.452394	-1.803602
14.	6.	0.	-2.258473	-1.280311	-2.108595
15.	6.	0.	-0.931664	-0.839196	-2.204670
16.	7.	0.	-0.625892	0.450255	-1.999732
17.	6.	0.	1.797503	-0.947190	-0.685228
18.	6.	0.	1.083363	-2.184374	-1.304580
19.	6.	0.	0.205173	-1.780217	-2.520502
20.	7.	0.	1.348703	-0.811131	0.599800

21.	6.	0.	0.481049	-1.857273	0.963244
22.	6.	0.	0.305772	-2.725470	-0.126100
23.	8.	0.	2.646565	-0.276659	-1.253640
24.	6.	0.	-0.135884	-2.080840	2.187904
25.	6.	0.	-0.939178	-3.223078	2.306878
26.	6.	0.	-1.115365	-4.102897	1.236033
27.	6.	0.	-0.491745	-3.852939	0.004470
28.	8.	0.	2.076994	-3.113616	-1.726894
29.	1.	0.	-0.771693	1.317676	3.767201
30.	1.	0.	1.579171	1.879488	4.306131
31.	1.	0.	3.062817	2.924719	2.603819
32.	1.	0.	-1.696906	1.749314	1.510000
33.	1.	0.	3.025676	3.818160	-0.090221
34.	1.	0.	1.424696	3.885070	-2.022012
35.	1.	0.	-3.746006	1.506695	-1.327692
36.	1.	0.	-1.126233	3.228780	-2.434429
37.	1.	0.	-2.139051	3.250464	-1.003089
38.	1.	0.	-2.509878	-2.323867	-2.282179
39.	1.	0.	0.867991	-1.304639	-3.251933
40.	1.	0.	-0.175619	-2.707143	-2.958917
41.	1.	0.	1.655875	-0.072956	1.221207
42.	1.	0.	0.000581	-1.398217	3.019532
43.	1.	0.	-1.431331	-3.424662	3.253537
44.	1.	0.	-1.738714	-4.983000	1.356378
45.	1.	0.	-0.627653	-4.534647	-0.829861
46.	1.	0.	2.720401	-2.605839	-2.248148

**1d**			Standard Orientation (Ångstroms)		
I	Atom	Type	X	Y	Z
1.	6.	0.	0.557755	5.656257	3.218827
2.	6.	0.	1.128507	4.972665	4.313849
3.	6.	0.	1.147862	3.582570	4.366154
4.	6.	0.	0.580531	2.886886	3.291870
5.	6.	0.	-0.002006	3.555253	2.176813
6.	6.	0.	-0.005281	4.962171	2.153787
7.	7.	0.	0.454687	1.534314	3.065509
8.	6.	0.	-0.183676	1.326149	1.858231
9.	6.	0.	-0.483111	2.536694	1.276376
10.	6.	0.	-3.258051	1.409700	-0.485489
11.	6.	0.	-1.870622	1.577640	-0.595379
12.	6.	0.	-1.160861	2.791058	-0.050205
13.	7.	0.	-3.884202	0.314557	-0.925958
14.	6.	0.	-3.120538	-0.634573	-1.478014
15.	6.	0.	-1.735720	-0.489859	-1.613057
16.	7.	0.	-1.127014	0.627001	-1.178284

17.	6.	0.	-0.059781	-2.565077	-0.044223
18.	6.	0.	0.382952	-1.933214	-1.407379
19.	6.	0.	-0.867329	-1.548999	-2.248610
20.	7.	0.	0.323828	-3.880724	-0.060126
21.	6.	0.	1.058602	-4.205463	-1.214686
22.	6.	0.	1.145534	-3.077534	-2.043169
23.	8.	0.	-0.640514	-1.997218	0.870887
24.	6.	0.	1.650209	-5.415515	-1.554440
25.	6.	0.	2.354694	-5.471170	-2.765271
26.	6.	0.	2.459361	-4.353549	-3.597017
27.	6.	0.	1.849229	-3.142763	-3.235993
28.	8.	0.	1.226038	-0.816442	-1.196444
29.	1.	0.	0.560529	6.742377	3.211259
30.	1.	0.	1.561015	5.542170	5.131250
31.	1.	0.	1.587012	3.055811	5.208155
32.	1.	0.	-0.440559	5.500332	1.316071
33.	1.	0.	0.765579	0.803517	3.686279
34.	1.	0.	-0.366342	0.319546	1.506320
35.	1.	0.	-3.870751	2.184139	-0.030401
36.	1.	0.	-0.426675	3.125520	-0.795148
37.	1.	0.	-1.883476	3.607697	0.060731
38.	1.	0.	-3.619272	-1.535754	-1.826146
39.	1.	0.	-0.492515	-1.178399	-3.210475
40.	1.	0.	-1.462449	-2.442267	-2.457167
41.	1.	0.	0.182725	-4.499903	0.726682
42.	1.	0.	1.576769	-6.283089	-0.907014
43.	1.	0.	2.829563	-6.403288	-3.056167
44.	1.	0.	3.016278	-4.421652	-4.525977
45.	1.	0.	1.932608	-2.269739	-3.876610
46.	1.	0.	0.630875	-0.046832	-1.060203

Figure S18. ^{1}H NMR spectrum of indolepyrazine B (**2**)

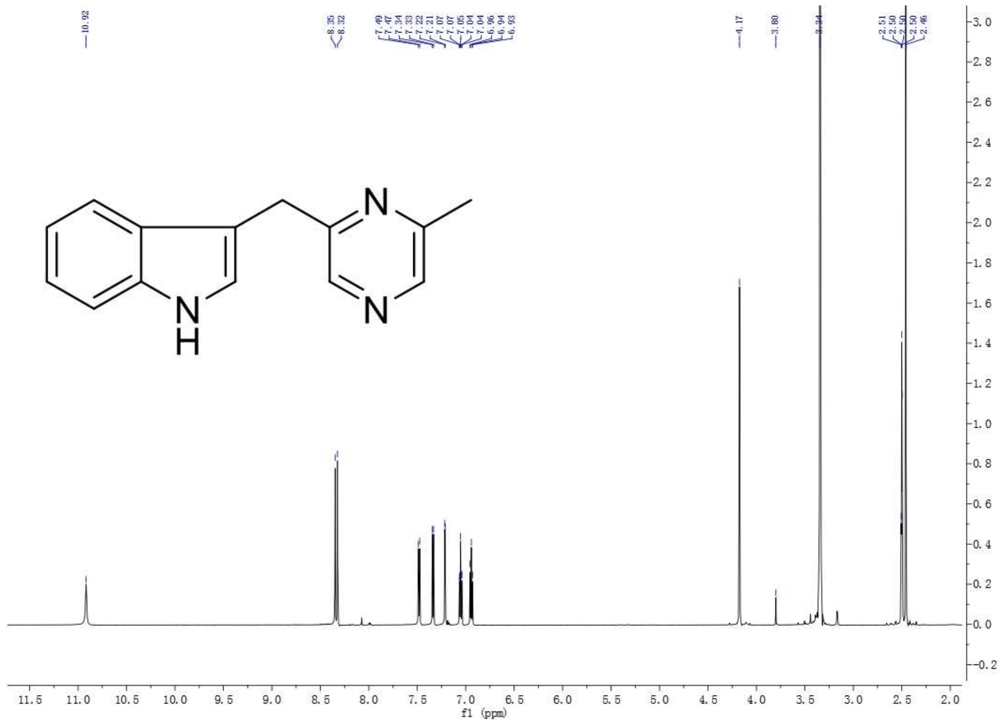

Figure S19. ^{1}H NMR spectrum of indolepyrazine B (**2**)

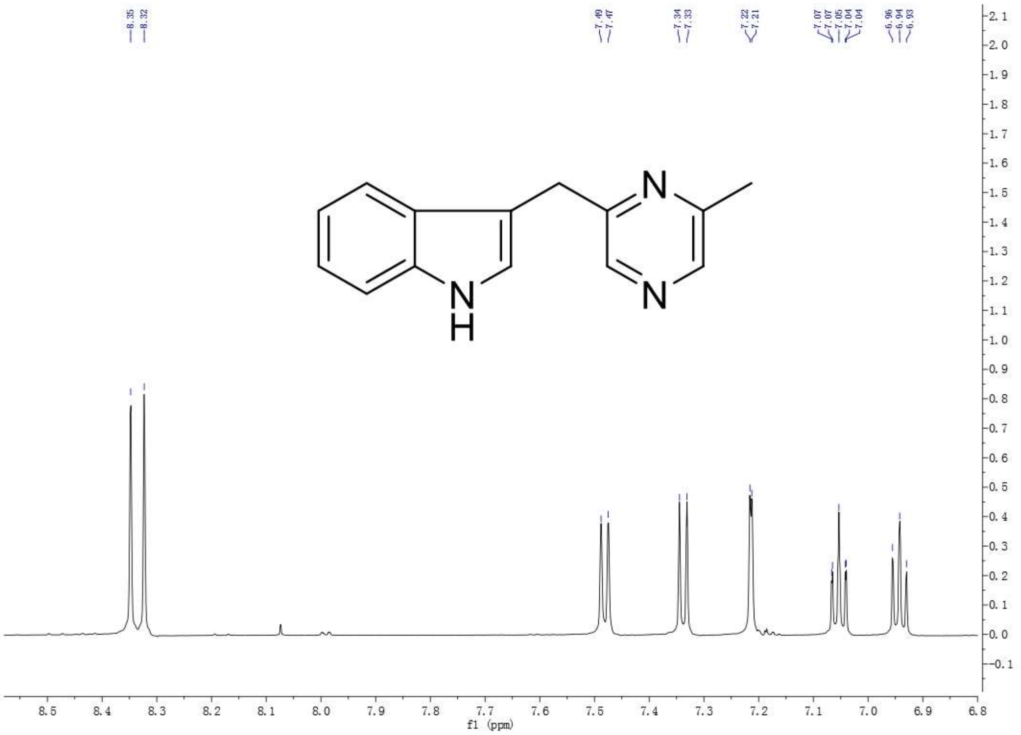

Figure S20. ^{1}H NMR spectrum of indolepyrazine B (**2**)

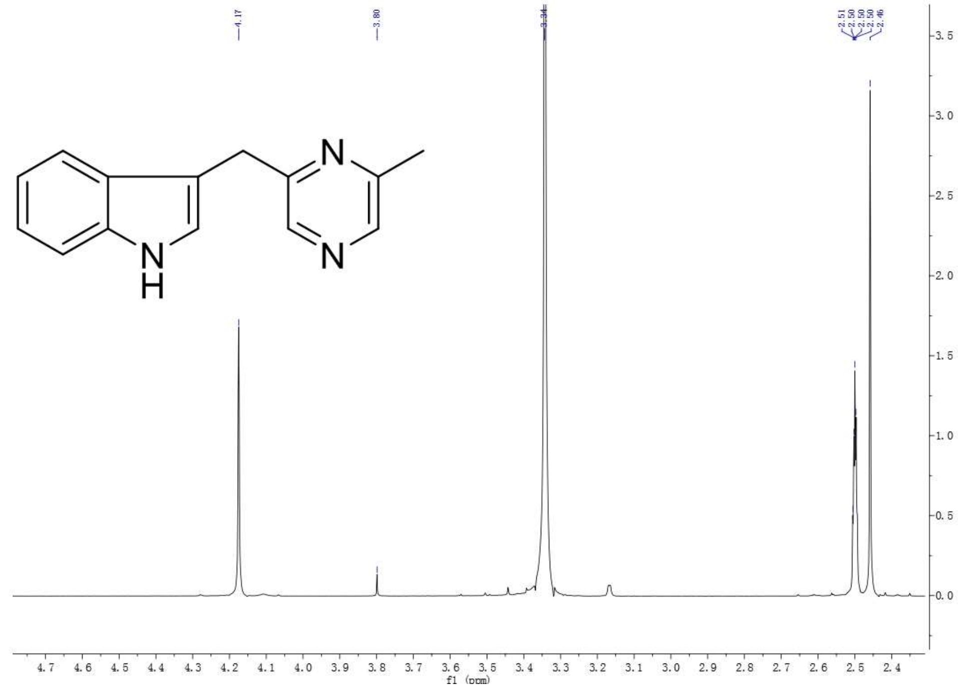

Figure S21. ^{13}C NMR spectrum of indolepyrazine B (**2**)

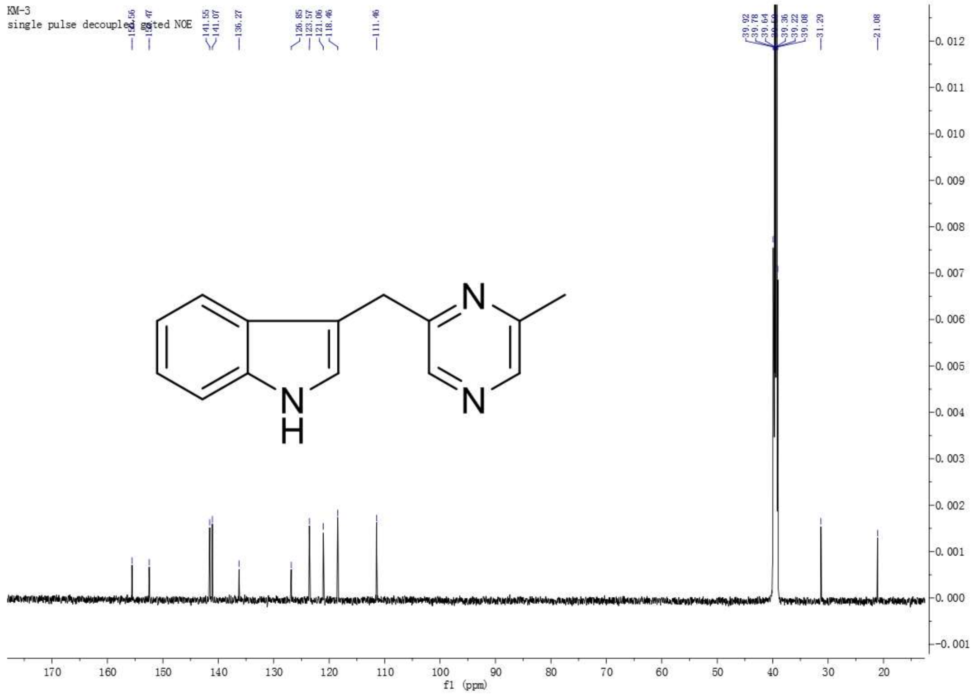

Figure S22. ^{13}C NMR spectrum of indolepyrazine B (**2**)

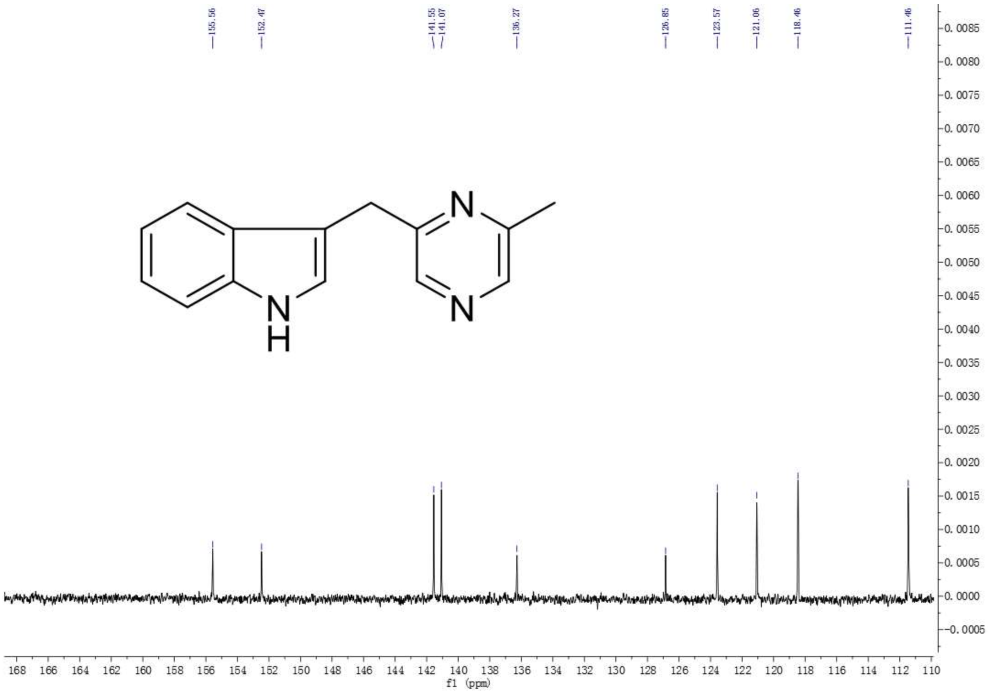

Figure S23. ^{1}H-^{1}H COSY spectrum of indolepyrazine B (**2**)

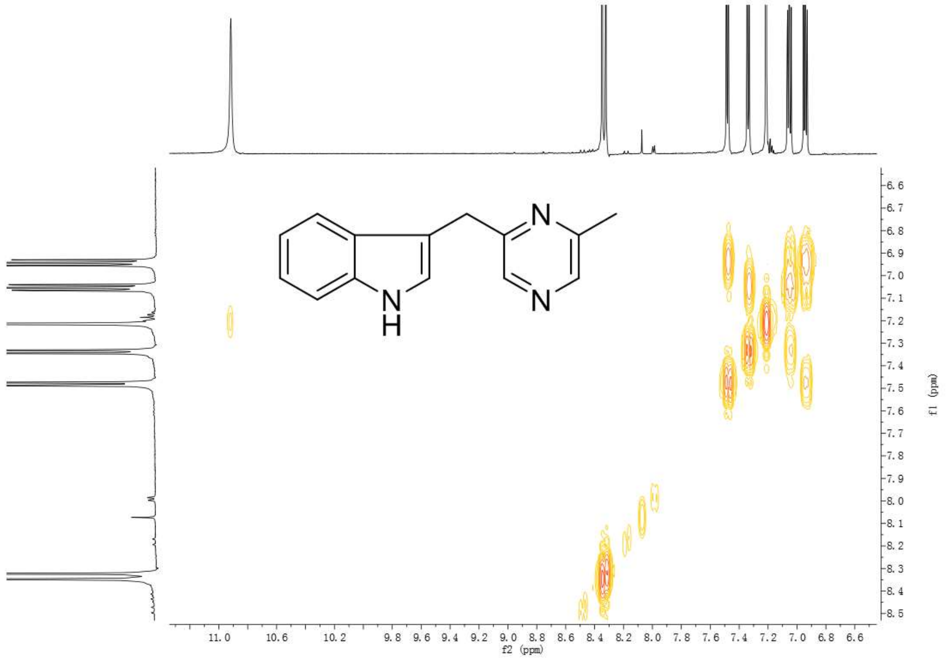

Figure S24. HSQC spectrum of indolepyrazine B (**2**)

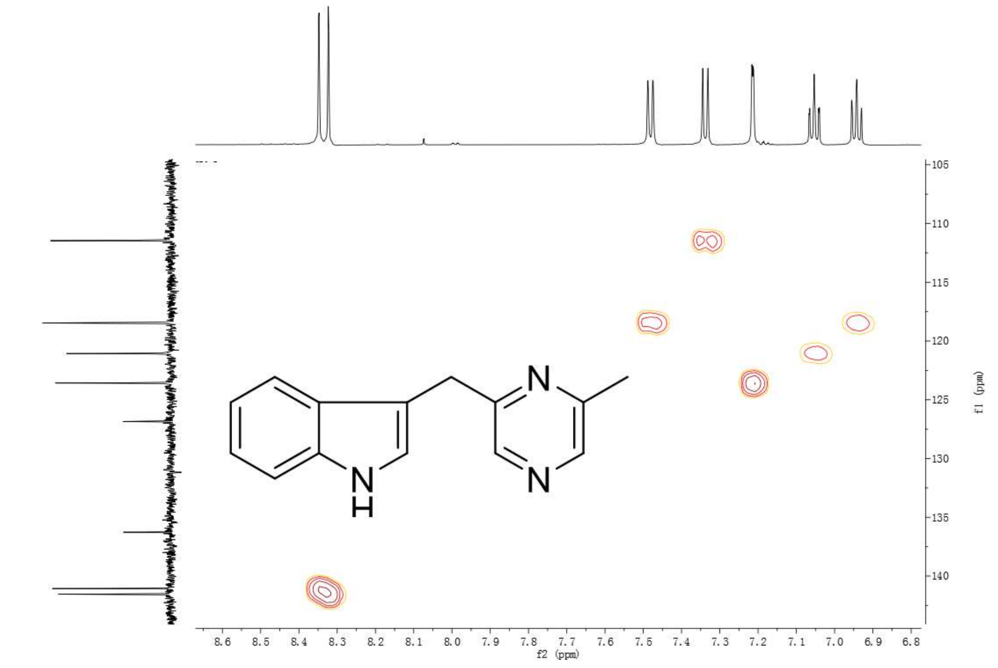

Figure S25. HSQC spectrum of indolepyrazine B (**2**)

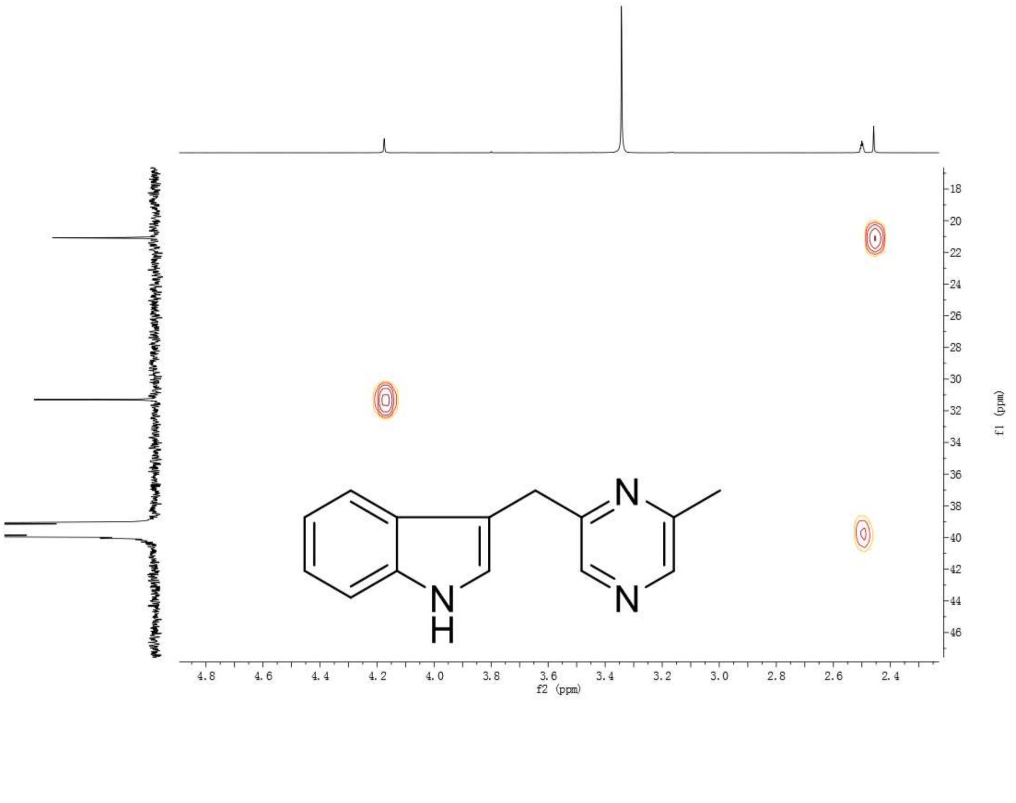

Figure S_{26}. HMBC spectrum of indolepyrazine B (**2**)

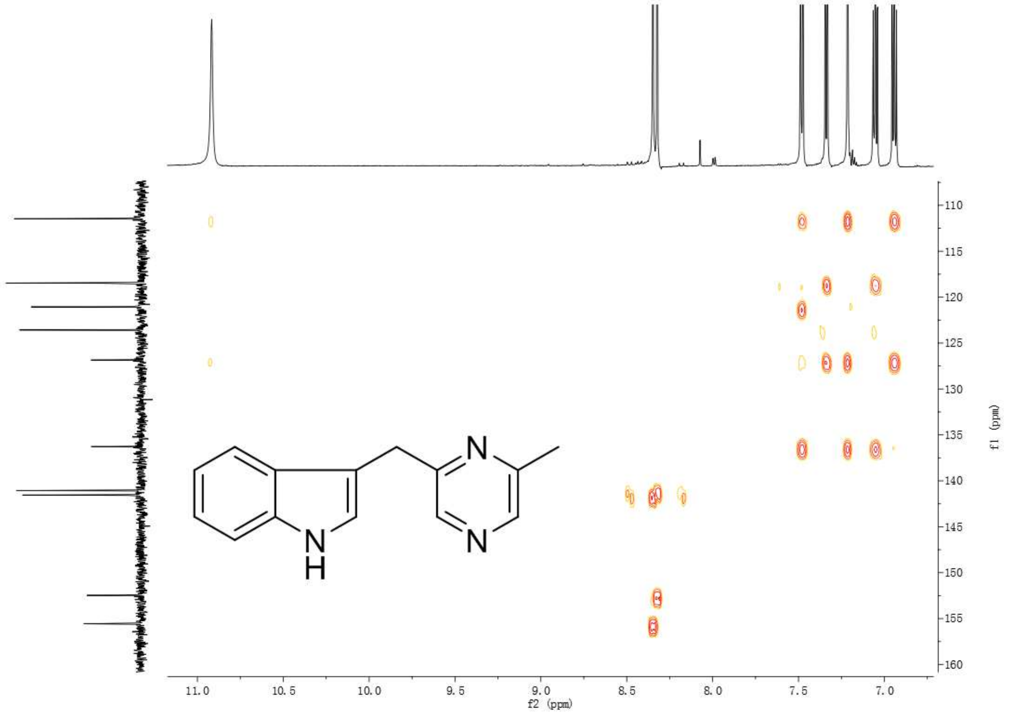

Figure S_{27}. HMBC spectrum of indolepyrazine B (**2**)

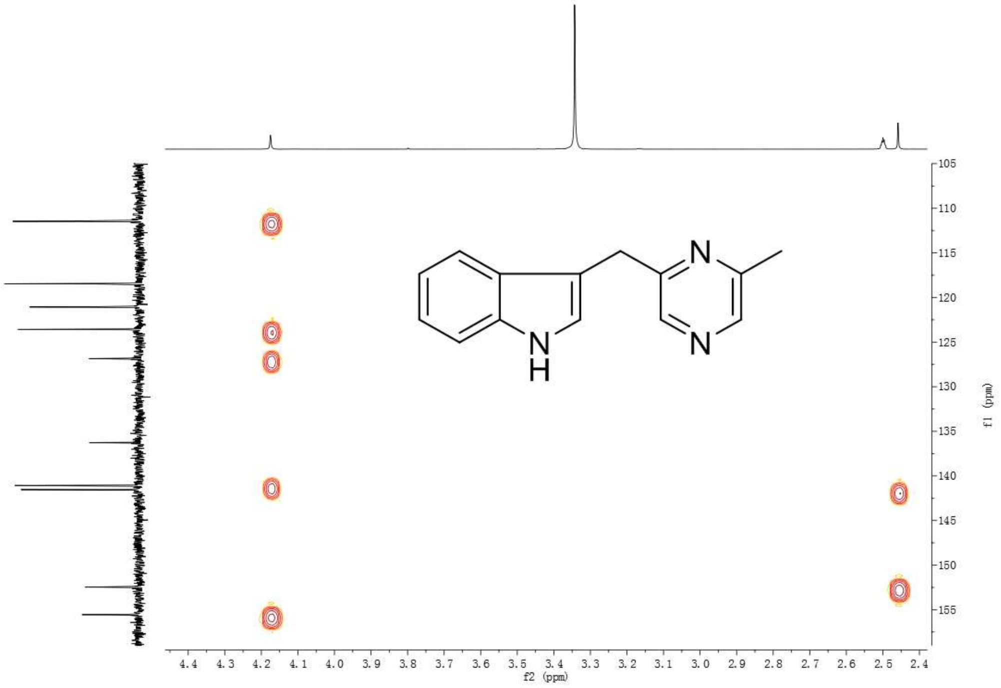

Figure S_{28}. HRESIMS spectrum of indolepyrazine B (**2**)

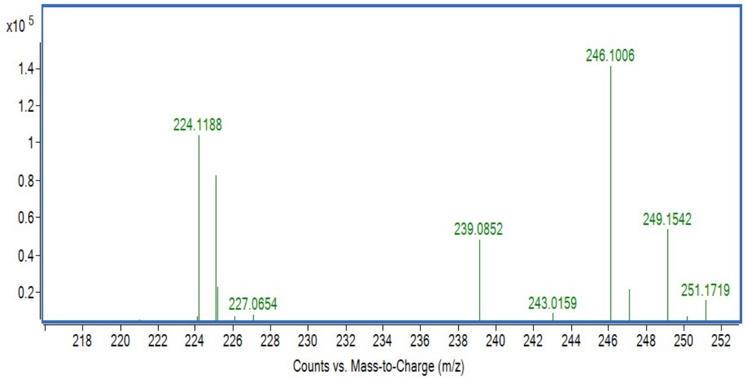

Figure S_{29}. UV spectrum of indolepyrazine B (**2**)

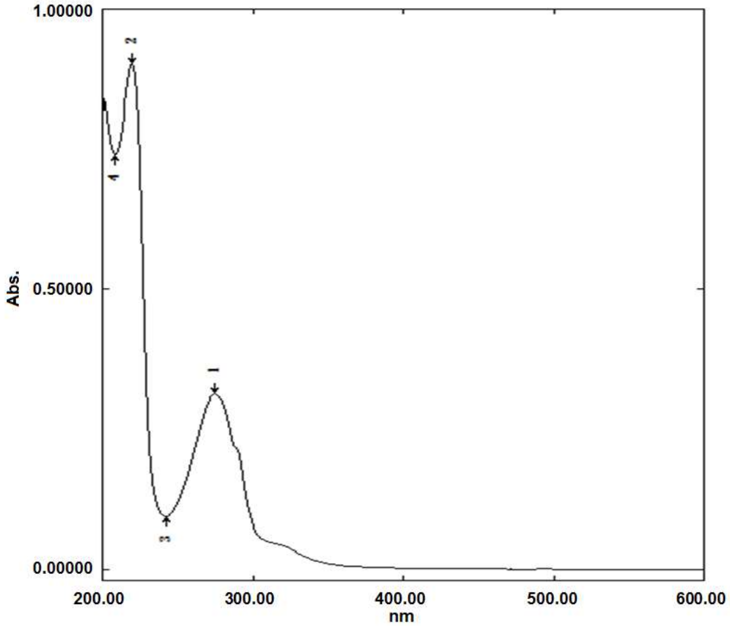

Supplement: Supplementary file 1 [file marinedrugs-17-00089-s001.pdf]
